# Microbial Cell Factory of Baccatin III Preparation in *Escherichia coli* by Increasing DBAT Thermostability and *in vivo* Acetyl-CoA Supply

**DOI:** 10.3389/fmicb.2021.803490

**Published:** 2022-01-12

**Authors:** Jia-jun Huang, Tao Wei, Zhi-wei Ye, Qian-wang Zheng, Bing-hua Jiang, Wen-feng Han, An-qi Ye, Pei-yun Han, Li-qiong Guo, Jun-fang Lin

**Affiliations:** ^1^Department of Bioengineering, College of Food Science, Institute of Food Biotechnology, South China Agricultural University, Guangzhou, China; ^2^Research Center for Micro-Ecological Agent Engineering and Technology of Guangdong Province, Guangzhou, China; ^3^Department of Pathology, Anatomy and Cell Biology, Thomas Jefferson University, Philadelphia, PA, United States

**Keywords:** baccatin III, 10-deacetylbaccatin III-10-*O*-transferase, gene integration, semi-rational design, thermostability, acetyl-CoA supplement

## Abstract

Given the rapid development of genome mining in this decade, the substrate channel of paclitaxel might be identified in the near future. A robust microbial cell factory with gene *dbat*, encoding a key rate-limiting enzyme 10-deacetylbaccatin III-10-*O*-transferase (DBAT) in paclitaxel biosynthesis to synthesize the precursor baccatin III, will lay out a promising foundation for paclitaxel *de novo* synthesis. Here, we integrated gene *dbat* into the wild-type *Escherichia coli* BW25113 to construct strain BWD01. Yet, it was relatively unstable in baccatin III synthesis. Mutant gene *dbat*^S189V^ with improved thermostability was screened out from a semi-rational mutation library of DBAT. When it was over-expressed in an engineered strain N05 with improved acetyl-CoA generation, combined with carbon source optimization of fermentation engineering, the production level of baccatin III was significantly increased. Using this combination, integrated strain N05S01 with mutant *dbat*^S189V^ achieved a 10.50-fold increase in baccatin III production compared with original strain BWD01. Our findings suggest that the combination of protein engineering and metabolic engineering will become a promising strategy for paclitaxel production.

## Introduction

Baccatin III is a precursor to paclitaxel (Taxol), a billion-dollar drug that is well known and recommended for treatment in more than 20 types of cancer (Wani et al., 1971; Cragg et al., 1993; Thornburg et al., 2017). Besides, this compound has been used to exert immunomodulatory activities on the major histocompatibility complex (MHC)-restricted antigen presentation (Lee et al., 2011), to slow the progression of various types of tumors (Lee et al., 2014), and to ameliorate bleomycin-induced pulmonary fibrosis (Nie et al., 2019). Baccatin III can be synthesized from 10-deacetylbaccatin III (10-DAB) by a one-step acyl transfer reaction of 10-deacetylbaccatin III 10-*O*-transferase (DBAT) (Walker and Croteau, 1999). Loncaric et al. (2006) verified the regiospecificity of DBAT by identifying the molecular structures of the products of 10-DAB and different coenzyme A (CoA) donors engaged in catalysis by this enzyme. The use of the robust DBAT and sufficient acetyl-CoA in a microbial cell factory provides an efficient way to produce baccatin III and avoids the low atom economy of its chemical synthesis (Danishefsky et al., 1996; Walker and Croteau, 2001).

DBAT is broadly distributed in plants (i.e., *Taxus* spp.) and endophytic fungi (Sah et al., 2019). Heterologous production of this eukaryotic protein in *Escherichia coli* may facilitate a higher yield of baccatin III because of (i) its unparalleled short generation times (Bentebibel et al., 2005; Loncaric et al., 2006; Malik et al., 2011), (ii) its higher cell intensity (Lee, 1996; Liu X. et al., 2018), and (iii) the use of a simpler required medium (Bonfill et al., 2007; Wang et al., 2016). Also, benefiting from the easy genetic manipulation tool of *E. coli*, a rapid gene integration process to further construct an engineered strain as a microbial cell factory is accessible (Wei et al., 2016). Li et al. (2017) expressed DBAT from six species of *Taxus* spp. in *E. coli* and discovered that the DBAT from *T. wallichiana* var. *mairei* showed the highest activity for 10-DAB. Although DBAT is highly regiospecific, these plant-derived genes generally have low thermal stability. Sadykhov et al. (2006) compared the thermal stability of formate dehydrogenases (FDH) from microorganisms and plants and found an inactivation rate constant for FDH from *Glycine max* (soybean) that was 10,000-fold higher than that for FDG from *Pseudomonas* sp. 101 at the same temperature (Alexandra et al., 1999). Thus, there is an urgent need to explore the thermostability of plant-origin DBATs when these are expressed *in vivo* in *E. coli*.

Semi-rational design by site-directed mutagenesis is a combination of directed evolution and rational design. Directed evolution is a useful tool to improve enzyme properties (Powell et al., 2001; Zhu and Zhang, 2017). However, it typically requires screening rare positive hits from huge volumes of mutants (Chica et al., 2005; Huang et al., 2019). The use of rational design can greatly decrease the size of the mutant libraries (e.g., down to hundreds of mutants), thus increasing the efficiency of the identification of desired mutants (Zhang et al., 2015; de Souza et al., 2016; Li et al., 2019). For example, Hotspot Wizard 3.0 (Sumbalova et al., 2018) is a web server for automatically established design of mutations and smart libraries in protein engineering, which is based on the amino acid frequency and evolutionary information from three databases^1^. Chen et al. (2018) applied homologous modeling, molecular docking, and the Hotspot Wizard 3.0 server to construct an L-rhamnose isomerase mutant with a 134.1% increase in relative activity when acting on D-allulose. Pongpamorn et al. (2019) developed a G513Y variant of flavin-dependent monooxygenase with an activity half-life that was 72-fold (50°C) and 160-fold (45°C) longer than the wild-type enzyme when assessed via the FireProt calculations (Musil et al., 2017) of the Hotspot Wizard 3.0 server. Semi-rational design is a suitable tool to investigate the thermostability of DBATs.

The natural substrates to generate baccatin III are 10-DAB and acetyl-CoA. Acetyl-CoA, a central metabolite, is involved in various biological processes (Krivoruchko et al., 2015) and acts as the acetyl donor in the biosynthesis of various acetyl chemicals (Lian et al., 2014). Modification of the acetyl-CoA metabolic pathway can provide efficient and rapid access to these metabolites (Liu et al., 2019; Zhang et al., 2019). Huang et al. (2018) constructed *E. coli* engineered strains to efficiently generate succinate from acetate via enhanced shunting of acetyl-CoA to succinate. Mainguet et al. (2013) reshaped a nonnative route for a glyoxylate shunt to allow the synthesis of four-carbon tricarboxylic acid (TCA) cycle intermediates from acetyl-CoA in *E. coli*, which can increase the carbon yield of acetate and biofuels from many carbon sources. Zhang et al. (2019) constructed *E. coli* metabolic engineered strains for efficient supply of acetyl-CoA from different carbon sources. Besides, studies suggest that when the products’ synthetic pathway involves metabolic intermediates, using glycerol as the fermentation carbon source is beneficial for products generation (Islam et al., 2017; Strucko et al., 2018; Yu et al., 2020). Hence, strengthening the accumulation and generation of acetyl-CoA by metabolic engineering or fermentation engineering is accessible and might provide significant amounts of acetyl donors to produce baccatin III.

In this study, two key factors, namely, DBAT activity and acetyl-CoA supplementation, were optimized to improve the production of baccatin III. The best single mutant of DBAT was screened out from a library of hotspot site-saturation mutants based on FireProt calculations with the Hotspot Wizard 3.0 server and the molecular mechanism was identified by thermostability investigation and molecular docking simulation analysis. Based on this, the engineered *E. coli* strain N05 with highly efficient supplied acetyl-CoA was used as a host and cultivated with an optimized carbon source to enhance the *in vivo* synthesis of baccatin III. The successful construction of an engineered *E. coli* strain N05S01 that yields high amounts of baccatin III provides a rapid and highly economical tool and lays out a foundation for the future industrial production of paclitaxel.

## Materials and Methods

### Strains, Reagents, and Chemicals

Strains and plasmids used in this study are listed in Table 1. The primers are listed in Supplementary Table 1. Strain *E. coli* N05, provided by Prof. Tao Yong of the Chinese Academy of Sciences, and *E. coli* BW25113 provided by Prof. Liu Jianzhong of Sun Yat-sen University, were used as the parental strain for gene integration and baccatin III production. PrimeSTAR^®^ Max DNA Polymerase, used for site-directed mutagenesis, was purchased from Takara Biomedical Technology Co., Ltd. (Dalian, China). Restriction enzymes and T4 DNA ligase were purchased from Thermo Fisher Scientific Co., Ltd. (Beijing, China). 10-DAB and baccatin III were purchased from J&K Scientific Co., Ltd. (Beijing, China). The HiPure Gel Pure DNA Micro Kit was acquired from Magen Co., Ltd. (Guangzhou, China). Acetyl-CoA was purchased from Sigma–Aldrich Inc. (St. Louis, MO, United States). A microorganism acetyl coenzyme A (ACA) enzyme-linked immunosorbent assay (ELISA) kit was obtained from Chundu Biotechnology Co., Ltd. (Wuhan, China). The general chemical reagents used in this study were obtained from standard suppliers.

**TABLE 1:** Strains and plasmids used in this study.

Strain/plasmid	Description	Source or reference
**Strains**		
*Escherichia coli*		
DH5α	F^–^ φ80 *lac* ZΔM15 Δ(*lac*ZYA-*arg* F) U169 *end*A1 *rec*A1 *hsd*R17(r_k_^–^,m_k_^+^) *sup*E44λ- *thi* -1 gyrA96 *rel*A1 *pho*A	Weidi Biotechnology Co., Ltd. (Shanghai, China)
DH5αλpir	F^–^ φ80 *lac* ZΔM15 Δ(*lac*ZYA-*arg* F) *LAMpir* U169 *end*A1 *rec*A1 *hsd*R17(r_k_^–^,m_k_^+^) *sup*E44λ- *thi* -1 *gyr*A96 *rel*A1 *pho*A	Provided by Prof. Liu Jianzhong of Sun Yat-sen University
BL21 (DE3)	F^–^ *omp*T *hsd*S_B_(r_B_^–^ m_B_^–^) *gal dcm*(DE3)	Weidi Biotechnology Co., Ltd. (Shanghai, China)
BW25113	*lacI*^q^ rrnB_T14_ Δ*lac*Z_WJ16_ *hsdR514* Δ*araBAD*_AH33_ Δ*rhaBAD*_LD78_	Provided by Prof. Liu Jianzhong of Sun Yat-sen University
BW25113 N05	*lacI*^q^ rrnB_T14_ Δ*lac*Z_WJ16_ *hsdR514* Δ*araBAD*_AH33_ Δ*rhaBAD*_LD78_ Δ*argB* Δ*argA* Δ*ptsG::glk* Δ*galR::zglf* Δ*poxB::acs* Δ*ldhA* Δ*gltA*	Provided by Prof. Tao Yong of the Chinese Academy of Sciences
BWD01	BW25113, *attP*_HK_::[P_T5_-*dbat*^o^]. Optimized gene *dbat* under the control of P_T5_ promoter being integrated into the genome of BW25113	This study
BWS01	BW25113, *attP*_HK_::[P_T5_-*s189v*^o^]. Optimized mutated gene *dbat*^S189V^ under the control of P_T5_ promoter being integrated into the genome of BW25113	This study
N05D01	N05, *attP*_HK_::[P_T5_-*dbat*^o^]. Optimized gene *dbat* under the control of P_T5_ promoter being integrated into the genome of N05	This study
N05S01	N05, *attP*_HK_::[P_T5_-*s189v*^o^]. Optimized mutated gene *dbat*^S189V^ under the control of P_T5_ promoter being integrated into the genome of N05	This study
**Plasmids**		
pET-32a-DBAT	Expression vector, pET-32a with fusion tags and gene *dbat* under P_T7_ promoter	Laboratory store
pET-32a-P_tac_-DBAT^S189V^	Expression vector, pET-32a with fusion tags and mutated gene *dbat*^S189V^ under dual promoter (P_T7_ and P_tac_)	This study
pCP20	pSC101 replicon^ts^ Flp(λR*p*) *cI*857, Cm^r^, Amp^r^	Provided by Prof. Liu Jianzhong of Sun Yat-sen University
pAH69	Helper plasmid expressing phage HK022 Int, Amp^r^	Provided by Prof. Liu Jianzhong of Sun Yat-sen University
pHKKT5b	Integration plasmid, *attP*_HK_ site P_T5_ promoter, Kan^r^	Provided by Prof. Liu Jianzhong of Sun Yat-sen University
pHKTT5b-*dbat*^o^	pHKTT5b derivative with the optimized gene *dbat* under the control of P_T5_ promoter	This study
pHKTT5b-*s189v*^o^	pHKTT5b derivative with the optimized mutated gene *dbat*^S189V^ under the control of P_T5_ promoter	This study

### Gene Integration and Fermentation

Gene *dbat* was amplified from the vector pET-32a-DBAT using the primers (see Supplementary Table 1) dbat_optF/rbs_dbat_optF/dbat_R to optimize the first 50 bp of gene *dbat* according to *E. coli* codon preference and adding RBS sequence in front of the optimized gene *dbat*. The method and PCR systems of gene integration are referred to Huang et al. (2014). The rbs_*dbat* fragment was cloned into the *Bam*HI/*Sal*I sites of pHKTT5b to obtain pHKTT5b-*dbat*^o^. The pHKTT5b-*dbat*^o^ was transformed into *E. coli* BW25113 and the vector integration was achieved with the help of the helper plasmid pAH69. The plasmid pCP20 was used to remove the FLP recognition target-flanked resistant marker in *E. coli* BW25113 (Lim et al., 2016). The engineered strain BWD01 integrating P_T5_ promoter and gene *dbat* in *E. coli* BW25113 was inoculated to a 250-mL shake-flask containing 50 mL fresh Terrific Broth (TB) medium (see Supplementary Table 2). 10-DAB with a final concentration of 80 μM was added as the substrate, and glycerol in TB medium acted as a carbon source. The bacteria were incubated at 150 rpm for 48 h at 37, 25, and 20°C, respectively.

### Analytical Methods

There was 1 mL of fermentation culture pipetted and centrifuged at 12,000 rpm for 5 min to separate the cell sedimentation and the medium supernatants. The baccatin III in the supernatants was analyzed using a high-performance liquid chromatography (HPLC) system [LC-2000, Techcomp (China) Ltd.] equipped with an ultraviolet (UV) detector (LC-2030) and a Diamosil C18 (2) column (250 mm × 4.6 mm, 5 μm). The mobile phase was 50% acetonitrile (A)-50% water (B), with a flow rate of 1 ml/min, at 28°C, and the UV detection wavelength was 227 nm. All assays were carried out in triplicate.

### Heterologous Expression of DBAT in *E. coli*

The pET-32a-DBAT was transformed into *E. coli* BL21 (DE3), then the recombinant was incubated in 50 ml fresh TB medium containing ampicillin (100 μg/ml) and incubated at 37°C with mixing at 200 rpm for 2–3 h until a spectrophotometer registered an optical density at 600 nm (OD_600_) of approximately 0.6–0.8. IPTG was added at a final concentration of 0.02 mM to induce gene expression, and the cell cultures were incubated for an additional 15 h at 20°C and 120 rpm. The following operations were performed on ice or at 4°C unless otherwise stated. After induction, cells were harvested by centrifugation (5,000 rpm, 4°C, 10 min) and washed twice with phosphate-buffered saline (PBS) [137 mM NaCl, 2.7 mM KCl, 10 mM Na_2_HPO_4_, and 2 mM KH_2_PO_4_, pH7.4]. The precipitates were stored at 4°C and were used for expression assays of DBAT and whole-cell catalysis.

The precipitates were resuspended in 10 ml lysis buffer [PBS buffer (pH 7.4), 1 mM PMSF, and 1 mM DTT], then sonicated for 12 min in an ice bath with 25% intermittent power (4 s on, 6 s off). The sample was centrifuged at 8,000 rpm at 4°C for 15 min to remove the cellular debris. The cell supernatants were filtered through a 0.22 μm membrane for further purification by using Ni-NTA agarose resin (Qiagen) according to the manufacturer’s manual. The crude extract, purified protein, and the cellular debris were detected by sodium dodecyl sulfate-polyacrylamide gel electrophoresis (SDS-PAGE) (12%) and Coomassie blue staining, respectively.

### Whole-Cell Catalysis System

The precipitates were resuspended in 2 mL of PBS, adjusted to an OD_600_ of 15. The cell mixtures were incubated in the presence of 80 μM 10-DAB and 2 mM glucose (acted as carbon source) at different temperatures (18°C–37°C) with 150 rpm shaking for 1 h to investigate the enzymatic function of DBAT. The reaction mixture was centrifuged at maximum speed for 10 min and the supernatant was analyzed by HPLC. For experimental controls, whole-cell biosynthesis (Wu and Li, 2018) of baccatin III without adding glucose was performed under the same conditions as already described. All assays were carried out in triplicate.

### Mutation Library Construction of DBAT

A three-dimensional (3D) structure model of DBAT was predicted by homology modeling via MODELLER 9.18 in our previous work (Lin et al., 2018). The model was uploaded to the Hotspot Wizard 3.0 online server, where the mutation hotspots and amino acid information in the solvent channel were predicted. Single-point mutations were determined by the Hotspot Wizard 3.0 server and generated via site-directed mutagenesis (details in Supplementary Material). The pairs of forward and reverse primers used are listed in Supplementary Table 3, with mutated bases in italics.

### Property Analysis of DBAT and Mutants

Wild-type DBAT (WT) strain and all mutant strains were induced in TB medium and sonicated as described. The crude extraction of all samples was detected by SDS-PAGE (12%) followed by Coomassie blue staining, and the expression level results were analyzed by the image analysis software Image J^2^ (Carvajal-Vergara et al., 2010; Liu Q. et al., 2018).

The best mutant from the library screening (DBAT^best^) and the WT strain were chosen for thermal stability analysis. The induction and expression were the same as described. The supernatants were used for the thermal stability study. The crude extraction of WT and the best mutant were incubated at 25°C for 0, 0.25, 0.5, 0.75, 1, 2, 3, 4, and 5 h, respectively. The assay mixture, which contained a certain amount of crude enzyme, 80 μM 10-DAB, 1 mM acetyl-CoA, and 1 mM Mg^2+^ in 50 mM PBS (pH 7.0), was allowed to react at 22°C for 30 min. The reaction was terminated by adding to the mixture 500 μl methanol and analysis was proceeded by HPLC. Relative conversion values were expressed as percentages relative to the conversion measured at the initial state (25°C, 0 h).

*In silico* studies were performed to analyze the difference between the skeleton of the mutation models and the WT model. Molecular docking studies were used to compare the conformation of the complex of DBAT^best^ with two substrates, 10-DAB and acetyl-CoA, and the complex of WT with the same substrates. Visual analysis used PyMOL software to investigate the change in the difference in the number of hydrogen bonds between both substrates and target proteins and the binding energy (a parameter showing stability) (Satish et al., 2018) of these two complexes.

### Mutant Gene Integration and *in vivo* Synthesis of Baccatin III

The DBAT^best^ gene was integrated into *E. coli* BW25113 via the same method as described, the engineered strain named BWS01. Fermentation of strain BWS01 was performed under the same condition as strain BWD01. Compared to the bioconversion capacity of producing baccatin III by engineered strains with different integrated genes to produce baccatin III.

### Improve the Supply of Acetyl-CoA to Enhance the Bioconversion Process

*Escherichia coli* strain N05 was reported as an engineered strain with high acetyl-CoA production. The DBAT^best^ gene was integrated into *E. coli* N05 via the same method as described, the engineered strain named N05S01. Fermentation of strain N05S01 was performed under the same condition as strain BWD01 and strain BWS01. Finally, to further improve the production of baccatin III, the concentration of glycerol in the TB medium was increased. After fermentation, baccatin III yields in culture supernatant were detected by HPLC as described. All assays were carried out in triplicate.

## Results

### Integration and Fermentation of the Engineered Strain BWD01

Engineered strain BWD01 was constructed with an integrant expression of *dbat* gene controlled by T5 promoter in *E. coli* BW25113 under the cooperation of the integration vector pHKTT5b, the helper plasmid pAH69, and pCP20. The supernatants of strain BWD01 fermented at 37°C showed no baccatin III in an HPLC analysis. However, the yield showed 4.68 ± 0.21 μM when the fermentation temperature was reduced to 25°C. Once the temperature was decreased to 20°C, the yield could reach 15.79 ± 0.63 μM. Results showed that the integrant expression of gene *dbat* in *E. coli* was relatively thermally sensitive.

### Heterologous Expression of DBAT and Whole-Cell Biosynthesis of Baccatin III

**FIGURE 1:**
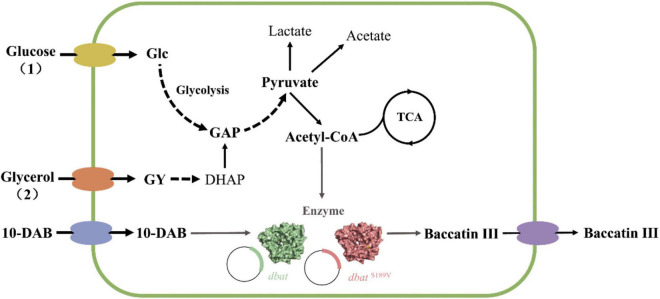
Process for whole-cell bioconversion of baccatin III by wild-type DBAT (WT) and DBAT^S189V^ in *E. coli*. **(1)** Producing baccatin III using glucose as a carbon source. Glucose is metabolized to produce pyruvate, which in turn, produces acetyl-CoA, which provides acetyl groups for 10-DAB to produce baccatin III. **(2)** Producing baccatin III using glycerol as a carbon source. Glycerol is metabolized to produce DHAP, then metabolized by glycolysis to produce pyruvate, which in turn, produces acetyl-CoA, which provides acetyl groups for 10-DAB, and produces baccatin III. The dotted arrow indicates that the multi-step reaction process is omitted in this metabolic pathway diagram. The gray arrow points to the direction of the enzymatic reaction to produce baccatin III. DBAT and DBAT^S189V^ were overexpressed in *E. coli* by recombinant plasmids. Glc glucose, GY glycerol; GAP, glyceraldehyde-3-phosphate; DHAP, dihydroxyacetone phosphate; 10-DAB, 10-deacetylbaccatin III; TAC, tricarboxylic acid cycle; DBAT, 10-deacetylbaccatin III-10-*O*-transferase.

Semi-rational design is a reliable strategy to improve the thermal stability of DBAT, and it is necessary to construct a heterologous plasmid expression system for investigating DBAT’s activity. DBAT expressed in *E. coli* BL21 (DE3) using the expression vector pET-32a partially formed inclusions. The protein purification showed that the purified protein located at a protein size of about 70 kDa was the 6 × His fusion target protein with a theoretical molecular weight of 67 kDa, which indicated the successful expression of DBAT in *E. coli* (Supplementary Figure 1). The recombinant cell pellet without sonication treatment was used for whole-cell biosynthesis of baccatin III. Using glucose as a carbon source substrate with 10-DAB addition, baccatin III can be detected in the supernatants after the reaction. Supplementary Figure 2 showed that without the participation of glucose, no product could be detected in the supernatant of the whole-cell system. It speculated that DBAT had little capacity to pull acetyl-CoA from the stable metabolic process. Results implied that glucose may act as an “activator” in this bioconversion to achieve the *in vivo* synthesis of baccatin III (Figure 1). The relative conversion of baccatin III presented a bell-shaped curve from 18 to 35°C and reached the maximum at 22°C (Supplementary Figure 3). The conversion of baccatin III at 25°C was the largest change, and it exactly decreased to 50%. When the temperature exceeds 35°C, no product could be detected. Therefore, 25°C, which allowed DBAT to present a medium level of conversion, was used as the subsequent screening temperature of the mutation library.

### Results of Mutation Library Screening

**FIGURE 2:**
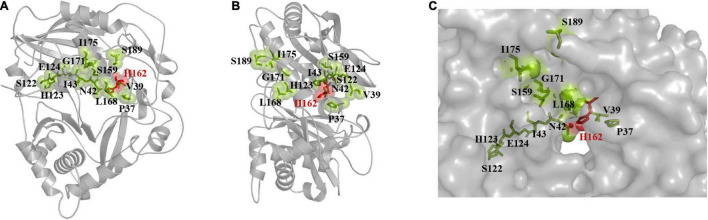
“Hotspots” distribution was predicted by Hotspot Wizard 3.0. The center residue His162 is displayed as dots style and shown in red. The others are displayed as spheres styles and shown in green by PyMOL. **(A)** The front face of the model is displayed as cartoon style. **(B)** The side face of the model is displayed as cartoon style. **(C)** The distribution of residues near the solvent channel, the model is displayed as surface style.

**FIGURE 3:**
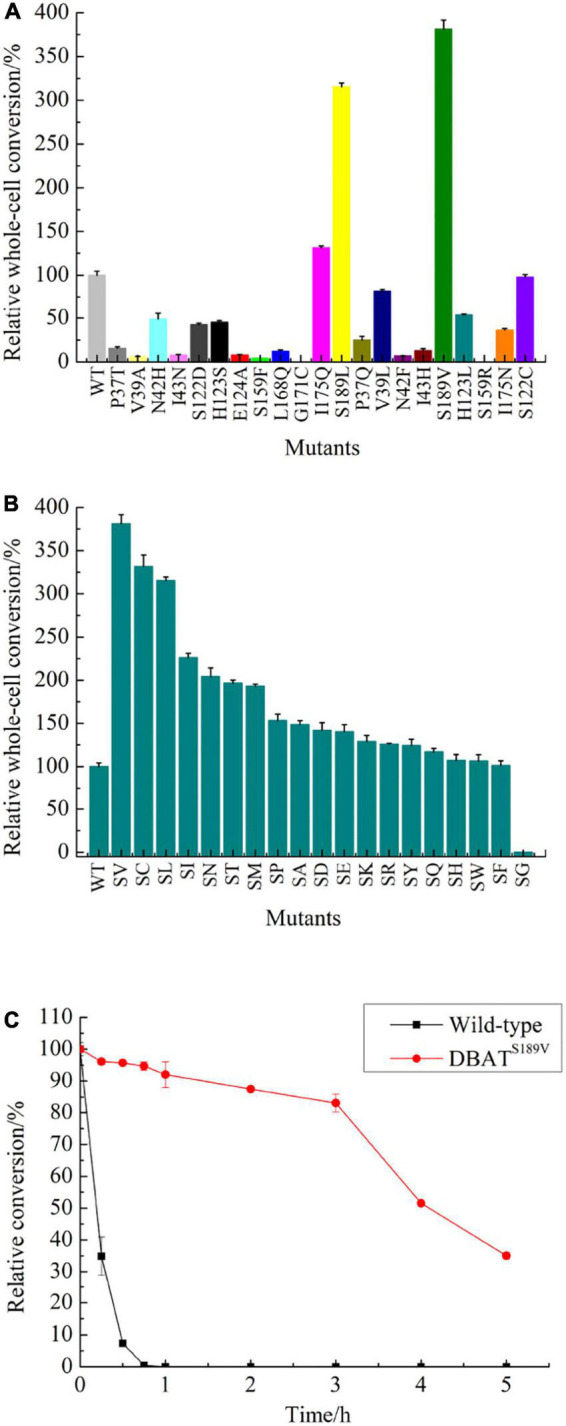
Two rounds of mutation library screening results and thermal stability analysis results. **(A)** Screening results for the “hotspot” mutants by whole-cell bioconversion. **(B)** Screening results for site-saturated mutations by whole-cell bioconversion. **(C)** Thermal stability of the crude enzymes of DBAT^S189V^ and WT.

The binding pocket and solvent channel of DBAT were predicted by the Hotspot Wizard 3.0 server (Supplementary Figure 4). There were 13 mutational “hotspots” (Pro37, Val39, Asn42, Ile43, Ser122, His162, His123, Glu124, Ser159, Leu168, Gly171, Ile175, and Ser189) in the solvent channel (Figure 2) chosen for site-directed mutagenesis. As the residue His162 was proved to be the key catalytic site of DBAT in our previous study (You et al., 2018). When the residue His162 was mutated to other amino acids, DBAT will completely lose its activity. Similar results were obtained in other studies (Li et al., 2017). Thus, the residue His162 was excluded in this study. Mutational landscapes, amino acid frequency, and the evolutionary information of these amino acid residues based on data from three major databases to provide mutation-selection were also analyzed by the server (see Supplementary Table 4). Each mutation acted on the production of baccatin III differently. Peak areas were converted to product concentrations based on external standard calibrations of baccatin III (Supplementary Figure 5). The yield of baccatin III obtained by the WT enzyme whole-cell catalytic reaction was used as the 100% whole-cell conversion rate of baccatin III. Compared with the yield of baccatin III, the mutant’s relative whole-cell conversion rate was obtained. Results showed that DBAT^G171C^ and DBAT^S159R^ had no measurable product in the whole-cell bioconversion of 10-DAB at 25°C (Figure 3A), while other mutations, such as DBAT^P37T^, DBAT^V39A^, DBAT^N42F^, and DBAT^H123L^, were negatively impacted on bioconversion. On the other hand, compared with that of WT, the bioconversion efficiency of DBAT^S189L^ and DBAT^S189V^ was relatively 3.15 and 3.81 times higher. Results suggested that residue Ser189 was probably the critical site for thermal stability improvement. To screen out the optimal point mutation of the Ser189 site, a new round of saturation mutation was performed. Results showed that DBAT^S189V^ still exhibited the best bioconversion ability of 10-DAB and DBAT^S189C^ was the second best with a 3.31-fold increase (Figure 3B). When S189 was mutated to leucine and isoleucine, the yields of baccatin III were also improved (higher than twofold). The remaining mutants were less effective in the catalytic activity.

### Expression, Stability, and *in silico* Analysis of Wild-Type and Mutants

Wild-type and all mutants were sonicated, and the supernatants were examined by SDS-PAGE (12%) followed by staining with Coomassie blue (Supplementary Figure 6). Located in the position of the mutant protein according to the WT target protein, and compared their protein expression levels roughly. No significant difference was shown in the expression levels of mutants and WT evaluated by Image J software (Supplementary Figure 7). The highest relative expression was found with DBAT^G171C^ and the lowest with DBAT^S159R^, both were negative mutants with unmeasurable products. DBAT^S189V^, the most effective mutant in biotransformation, was only 88.09% of the WT. Results suggested that the soluble expression level might not be the key factor in the semi-rational evolution of DBAT.

Thermal stability results showed that DBAT^S189V^ could still retain 80% of its activity when placed at 25°C for 3 h. Its *t*_1/2_ value (half-life period) at 25°C was about 245 min. In contrast, the *t*_1/2_ value of WT was only about 10 min (Figure 3C), and its activity was lost, and the product was undetectable after 1 h. The thermal stability analysis results showed that the half-life of mutant S189V at 25°C was 24.5 times longer than that of WT.

**FIGURE 4:**
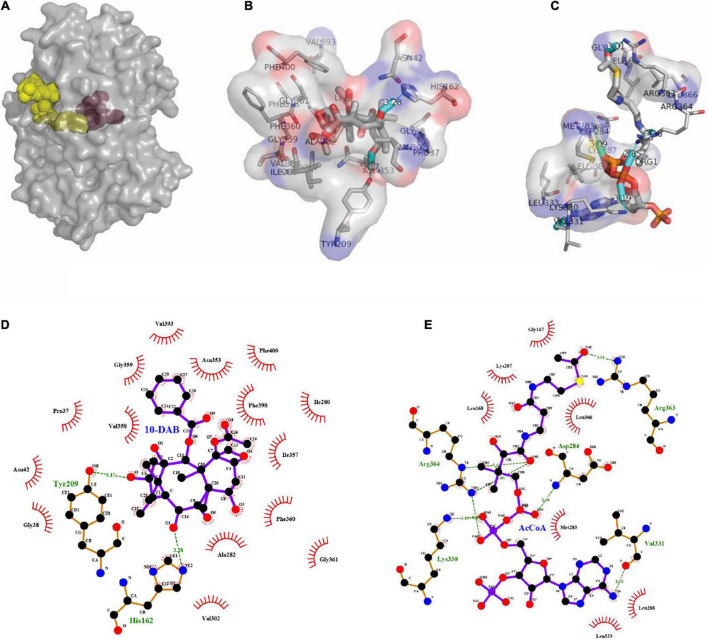
Molecular docking results of DBAT^S198V^ and two substrates. The complex was named S189V_10-DAB_AcCoA. **(A)** Panoramic conformation of S189V_10-DAB_AcCoA. **(B)** Interaction between 10-DAB and DBAT^S189V^ in 3D-model. **(C)** Interaction between acetyl-CoA and DBAT^S189V^ in 3D-model. **(D)** The hydrogen bonds were shown as green dotted lines between 10-DAB and DBAT^S189V^. **(E)** The hydrogen bonds were shown as green dotted lines between acetyl-CoA and DBAT^S189V^.

**FIGURE 5:**
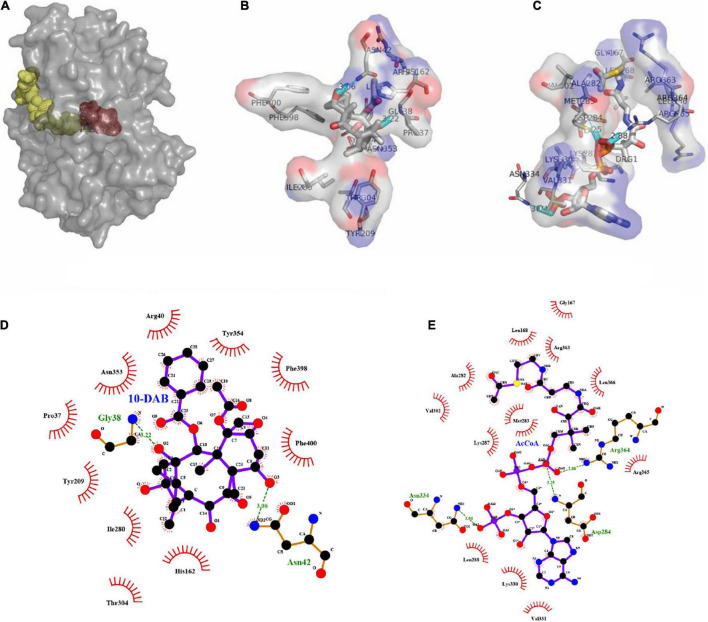
Molecular docking results of WT and two substrates. The complex was named WT_10-DAB_AcCoA. **(A)** Panoramic conformation of WT_10-DAB_AcCoA. **(B)** Interaction between 10-DAB and WT in 3D-model. **(C)** Interaction between acetyl-CoA and WT in 3D-model. **(D)** The hydrogen bonds were shown as green dotted lines between 10-DAB and WT. **(E)** The hydrogen bonds were shown as green dotted lines between acetyl-CoA and WT.

As a result of molecular docking, it could also be seen that two substrates, namely, 10-DAB and acetyl-CoA, entered the target protein from both sides, with mutual binding of the target protein in the solvent channel (Figures 4A, 5A). On one hand, it was found that the binding energy of the mutational complex S189V_10-DAB_AcCoA (S189V docking with acetyl-CoA and 10-DAB) was lower than that of the wild-type complex WT_10-DAB_AcCoA (WT docking with acetyl-CoA and 10-DAB), which was reflected in its improved stability (Table 2). On the other hand, the number of hydrogen bonds formed between substrates and DBAT^S189V^ (Figures 4B–E) was more than that between substrates and WT (Figures 5B–E). All the results further indicated that the conversion from serine to valine improved the structural and thermal stability of DBAT and consequently increased production.

**TABLE 2:** Key parameters of docking complex WT_10-DAB_AcCoA and S189V_10-DAB_AcCoA.

	WT	DBAT^S189V^
Binding energy with 10-DAB (kj/mol)	−8.7	−9.4
Binding energy with acetyl-CoA (kj/mol)	−7.8	−8.3
Numbers of hydrogen bonds with 10-DAB	2	2
Numbers of hydrogen bonds with acetyl-CoA	3	7

### The Effect of Carbon Source Optimization on the Synthesis of Baccatin III

**FIGURE 6:**
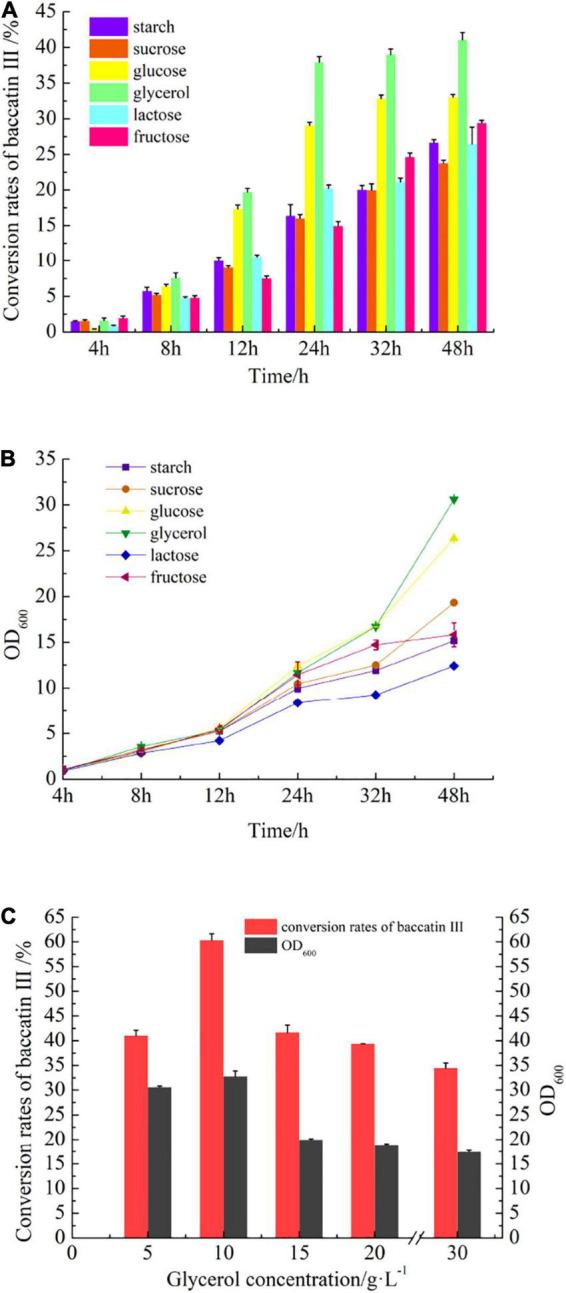
The effect of carbon source optimization on baccatin III production and cell growth during fermentation. **(A)** The effect of different carbon sources on baccatin III production. **(B)** The effect of different carbon sources on cell growth. Take OD_600_ value as the parameter of cell concentration. **(C)** The effect of glycerol content on baccatin III production and cell growth.

Glycerol was screened out in carbon source optimization from starch, sucrose, glucose, glycerol, lactose, and fructose (Figure 6A, details in Supplementary Material). Fermentation with glycerol (Figure 1) in the same concentration, the conversion rate of baccatin III could reach 41.05 ± 1.06% (increased by 24.3% compared with glucose). Interestingly, the cell density of the fermentation broth also increased and OD_600_ reached 30.6 (increased by 16.2% compared with glucose) (Figure 6B). The addition of a carbon source was continuously increased, and it was found that when glycerol was added to a concentration of 10 g/L, the conversion rate of baccatin III could reach a maximum of 60.36 ± 1.29%, and the OD_600_ could reach 32.78 (Figure 6C).

### The Effect of the Optimized Strain of Acetyl-CoA Supplementation on the Yield of Baccatin III

**FIGURE 7:**
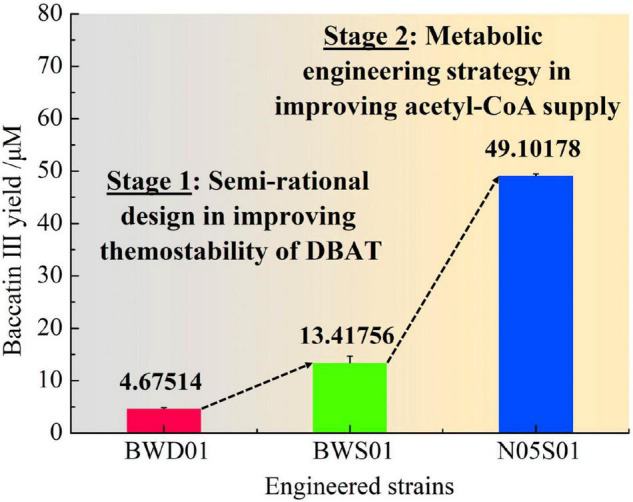
Results of the fermentation of integrated engineered strains to produce baccatin III. Baccatin III production has undergone two stages of improvement. Stage 1, Semi-rational design in improving thermostability of DBAT; Stage 2, Metabolic engineering strategy in improving acetyl-CoA supply.

Strain N05 is a metabolically engineered strain that can efficiently accumulate acetyl-CoA (Zhang et al., 2019). The mutant gene *dbat*^S189V^ was integrated into the chromosomes of *E. coli* strains BW25113 and N05, respectively. Two engineered strains, BWS01 (BW25113::*dbat*^S189V^) and N05S01 (N05::*dbat*^S189V^) were successfully constructed. Using 10-DAB with a final concentration of 80 μM, baccatin III production in the culture supernatant of strain N05S01, strain BWS01, and strain BWD01 fermented at 25°C for 48 h in the optimal condition of using glycerol as the carbon source with 10 g/L concentration were detected (Figure 7). The yield of baccatin III of strain N05S01 was 49.10 ± 0.36 μM (28.8 mg/L), which was 10.50 times higher than that of the fermentation product of strain BWD01 (4.68 ± 0.21 μM).

## Discussion

Since the *dbat* gene was successfully cloned over two decades, research on DBAT has mainly focused on (i) cloning and heterologous expression of DBAT from different species (Guo et al., 2007; Han et al., 2014; Sah et al., 2019), (ii) the regional specificity and substrate diversity of DBAT (Loncaric et al., 2006, 2007), and (iii) the exploration of the unnatural substrate (Li et al., 2017; Lin et al., 2018; You et al., 2018, 2019; Huang et al., 2020). However, research on the thermal stability of DBAT has not been reported. In this study, baccatin III cannot be detected in the fermentation supernatant of the *dbat* gene-integrated strain BWD01 fermented at 37°C. It was found that the induction temperature of DBAT was mainly low (probably 20°C or 16°C) in the studies of the heterologous expression of DBAT in *E. coli* (Li et al., 2017; Lin et al., 2018). In this study, fermentation temperature increased from 20°C to 25°C and reduced 70.36% of the baccatin III yield. Taken together, improving the thermal stability of DBAT of plant origin might be a considerable path to increase the yield and titer of baccatin III in the *E. coli* cell factories (Huang et al., 2020).

In the following study, semi-rational evolution was applied to improve the thermal stability of DBAT. S189, which was located at the entrance of the solvent channel as well as the surface of DBAT, was the crucial residue selected by the mutation library in this study. Compared with the WT, the yield of baccatin III produced by mutants DBAT^S189V^, DBAT^S189C^, DBAT^S189I^, and DBAT^S189L^ were significantly increased. When the serine at position 189 was mutated to cysteine, the surface structure of mutant DBAT^S189C^ changed, and a hydrogen bond was formed between Cys189 and Lys295, which is also located on the surface structure of the enzyme (Supplementary Figure 8) and might enhance the stability of the structure (Sun et al., 2015; Muchiri and Walker, 2017). Therefore, it was speculated that the S189C mutation improves the catalytic efficiency of DBAT by enhancing structural stability. DBAT^S189V^, DBAT^S189I^, and DBAT^S189L^ were mutants that represented a transition from a hydrophilic amino acid to a hydrophobic amino acid. Changing S189 into a hydrophobic amino acid was conducive to creating a hydrophobic environment that was more suitable for substrate entry and reaction. Gallardo et al. (2010) reported that increasing the hydrophobic folding of the enzyme near the binding pocket was beneficial in terms of improving its stability. Meanwhile, studies showed that valine located on the surface of the enzyme plays an important role in the stability of protein structure (Sugita et al., 1998; Wu et al., 2009; Weisslocker-Schaetzel et al., 2017). Valine, leucine, and isoleucine presented similar molecular structures and hydrophobic effects on DBAT. Valine is not only a hydrophobic amino acid, but also plays an important role in improving the rigidity of enzymes, and it can also form hydrogen bonds with Lys295 (Supplementary Figures 8E,F), which might explain why DBAT^S189V^ has the best catalytic effect.

Enhancing the stability of the enzyme surface and the hydrophobicity of the entrance of the enzyme activity channel have a significant effect on improving the catalytic efficiency of the enzyme. Recently, it was reported that enhancing the subunit interface of enzymes can improve stability more efficiently. Computational interface redesign can be a rapid and powerful strategy for enzyme stabilization (Meng et al., 2020).

Through the optimization of carbon source types, it was found that glycerol was the most suitable carbon source for baccatin III production. Discussed from the perspective of the metabolic process after the carbon source enters the strain, all kinds of carbon sources generate pyruvate through the glycolysis pathway and then form acetyl-CoA to provide raw materials for the reaction. Starch, sucrose, lactose, and fructose all need to produce glucose before entering the glycolysis pathway. Therefore, the path to produce acetyl-CoA is longer than the metabolic path of glucose. However, fructose has a shunt path that can enter the glycolysis pathway through generating fructose 6-phosphate. Thus, the effect of fructose was relatively better than the other three. Glycerol can enter the glycolysis pathway by forming glyceraldehyde 3-phosphate (GAP) in three steps. It was shorter than that from glucose (four steps). Therefore, glycerol became the most suitable carbon source in the pathway to generate acetyl-CoA, glucose and fructose were followed by Figure 6A. Through the optimization of carbon source dosage, it was also found that the supply of intracellular acetyl-CoA was insufficient for DBAT^S189V^ (Figure 6C). To investigate the acetyl-CoA accumulation after generating pyruvate from the glycolysis pathway is necessary. Zhang et al. (2019) constructed an engineered *E. coli* strain that modified the metabolic pathway of the metabolic node substance acetyl-CoA genetically can efficiently be synthesized *N*-acetylglycine using different carbon sources. Strain N05 has knocked out the *gltA* gene, a downstream gene of the metabolism of acetyl-CoA, to block acetyl-CoA from entering the tricarboxylic acid cycle, which enhanced the accumulation of acetyl-CoA in the result.

Testing by a microorganism acetyl coenzyme A (ACA) enzyme-linked immunosorbent assay (ELISA) kit (Chundu Biotechnology Co., Ltd.), the content of acetyl-CoA in BL21 (DE3) decreased during the test period (OD_600_ value varied from 0.6 to 1.6). In contrast, the knockout of gene *gltA* leads to the increase of acetyl-CoA accumulation in strain N05 during the logarithmic phase (Supplementary Table 5). The shunting of acetyl-CoA flux contributed more to baccatin III production. The engineered strain with gene *gltA* deletion might establish a better balance between cell growth and baccatin III production.

Currently, the preparation method of baccatin III is mainly through direct extraction from yews. The yield of baccatin III obtained by using plant cell *in vitro* culture preparation is low (2.4–4.6 mg/L by using immobilized cell culture in shaking flask and 7.8 mg/L by using bioreactor) (Malik et al., 2011). The upgrade of the bioreactor and the addition of different types of elicitors and inducers had produced a corresponding increase in the production of baccatin III by suspension cell culture, but they also take 8 days to 1 month for preparation. Compared to the methods mentioned above, microbial cell factory with integrant thermal stable mutant expression in this study is marker-less, short-period, and highly efficient. It could consume a simple carbon source and require no additional cofactor in fermentation. Considering the high medicinal value of baccatin III and paclitaxel, the engineered strains constructed in this study provided a competitive way for industrial synthesis.

## Conclusion

Given that the wild-type DBAT was thermally unstable, semi-rational evolution of DBAT was performed in this study. Bioinformatics methods (e.g., HotSpot Wizard, and Molecular Docking) were carried out and 13 mutation sites were screened. The conversion efficiency of mutant DBAT^S189V^, the best candidate, was higher than that of wild-type DBAT (a 3.81-fold increase). In addition, to meet the increasing demand of acetyl donors from mutant DBAT, *E. coli* strain N05, which could generate more acetyl-CoA, was used as a host for integrant expression of mutant DBAT^S189V^. The engineered strain N05S01 produced 49.10 ± 0.36 μM baccatin III after fermentation at 25°C for 48 h. It was 10.50 times higher than that of strain BWD01, which had no optimization in thermal stability of DBAT and acetyl-CoA supply. Taken together, this research combined the approaches of protein engineering and metabolic engineering to construct a cell factory with an increased yield of the exogenous products baccatin III. It laid out a valuable foundation for the construction of the paclitaxel cell factory in the coming future.

## Data Availability Statement

The original contributions presented in the study are included in the article/Supplementary Material, further inquiries can be directed to the corresponding author/s.

## Author Contributions

J-JH and TW carried out the main work, collected and analyzed the data, and drafted the manuscript. W-FH, A-QY, and P-YH participated in the research. Z-WY, Q-WZ, and B-HJ supervised the work, participated in data analysis, and revised the manuscript. J-FL and L-QG participated in the conception and design of the study, and finalized the manuscript. All authors read and approved the final manuscript.

## Conflict of Interest

The authors declare that the research was conducted in the absence of any commercial or financial relationships that could be construed as a potential conflict of interest.

## Publisher’s Note

All claims expressed in this article are solely those of the authors and do not necessarily represent those of their affiliated organizations, or those of the publisher, the editors and the reviewers. Any product that may be evaluated in this article, or claim that may be made by its manufacturer, is not guaranteed or endorsed by the publisher.

## Footnotes

1
https://loschmidt.chemi.muni.cz/hotspotwizard

2
http://rsb.info.nih.gov/ij

## Abbreviations

DBAT: 10-deacetylbaccatin III 10-*O*-transferase
10-DAB: 10-deacetylbaccatin III
WT: wild-type DBAT
IPTG: isopropyl- β-D -thiogalactopyranoside
ACA ELISA: acetyl-coenzyme A enzyme-linked immunosorbent assay
AcCoA: acetyl-coenzyme A
LB: Luria-Bertani
TB: Terrific Broth
TB-10-gly: TB medium with glycerol at a concentration of 10 g/L
SDS-PAGE: sodium dodecyl sulfate-polyacrylamide gel electrophoresis
HPLC: high-performance liquid chromatography.
