# Supplementary material for: Microbial Cell Factory of Baccatin III Preparation in Escherichia coli by Increasing DBAT Thermostability and in vivo Acetyl-CoA Supply
Source: Front Microbiol. 2022 Jan 12;12:803490. doi: 10.3389/fmicb.2021.803490 (PMC8790024; doi:10.3389/fmicb.2021.803490)
Supplement: Supplementary file 1 [file Data_Sheet_1.docx]

**Supplementary materials**

**Microbial Cell Factory of Baccatin Ⅲ Preparation in *Escherichia coli* by Increasing DBAT Thermostability and *in vivo*Acetyl-CoA Supply**

**Jia-jun Huang^1,3,#^, Tao Wei^1,3,#^, Zhi-wei Ye^1,3^, Qian-wang Zheng^1,3^, Bing-hua Jiang^2^, Wen-feng Han^1,3^, An-qi Ye****^1,3^, Pei-yun Han^1,3^, Li-qiong Guo^1,3,*^ , Jun-fang Lin^1,3,*^**

^1^Department of Bioengineering, College of Food Science and Institute of Food Biotechnology, South China Agricultural University, Guangzhou 510640, China

^2^Department of Pathology, Anatomy and Cell Biology, Thomas Jefferson University, Philadelphia, PA19107, USA

^3^Research Center for Micro-Ecological Agent Engineering and Technology of Guangdong Province. Guangzhou 510640, China

# Jia-jun Huang and Tao Wei are co-first authors. They contributed equally to this work.

***Correspondence:**

Corresponding Author: Professor Jun-fang Lin and professor Li-qiong Guo.

E-mails: linjf@scau.edu.cn (J.F.L.) and guolq@scau.edu.cn (L.Q.G.).

| **Fig. S1.** | SDS-PAGE analysis of DBAT expression and purification. The target protein DBAT is about 67 kDa.**M** molecular weight markers, **1** precipitation after cell disruption, **2** crude extract,**3-8** filtrate of the purification process, **9-11** purified protein. |
| --- | --- |
| **Fig. S2.** | The HPLC results of whole-cell bioconversion. **(A**) HPLC result for standard baccatin Ⅲ. (**B**) The results of whole-cell bioconversion to baccatin Ⅲ by adding glucose as an activator. (**C**) The results of whole-cell bioconversion to baccatin Ⅲ without adding glucose. |
| **Fig. S3.** | Theeffect of temperature on the production of baccatin Ⅲ by whole-cell bioconversion. |
| **Fig. S4** | The solvent channel and binding pocket of DBAT. |
| **Fig. S5.** | The baccatin Ⅲ standard curve. |
| **Fig. S6** | The SDS-PAGE results of mutants by staining with Coomassie blue.(**A, B)**SDS-PAGE analysis of “hotspot” mutants, 1-21: P37T,V39A, N42H,I43N,S122D,H123S,E124A,S159F,L168Q,G171C,I175Q,S189L,P37Q,V39L,N42F,I43H,S189V,H123L,S159R,I175N, and S122C. (**C, D**) SDS-PAGE analysis of site-saturated mutants. The band with the black arrow represents the target protein. |
| **Fig. S7.** | Relative supernatant expression of mutants. (**A**) The expression level results for“hotspot” mutants. (**B**) The expression level results forsite-saturated mutants. |
| **Fig. S8** | Skeleton structure analysis of the S189 mutants.**(A, B)**The distance between Ser189 and the adjacent residue Lys295 in WT is 4.8 Å. The DBAT model was displayed as cartoon style in green, the residue Lys295 and Ser189 were displayed as sticks style in blue. **(C, D)**The hydrogen bond between Cys189 and the adjacent residue Lys295 in DBAT^S189C^, the distance between them is 2.8 Å. The DBAT^S189C^ model was displayed as cartoon style in red, the residue Lys295 and Cys189 were displayed as sticks style in yellow.**(E, F)** The hydrogen bond between Val189 and the adjacent residue Lys295 in DBAT^S189V^, the distance between them is 2.4 Å. The DBAT^S189V^ model was displayed as cartoon style in yellow, the residue Lys295 and Val189 were displayed as sticks style in purple. |
| **Table S1** | Primers used for vectors construction. |
| **Table S2** | Formulation of medium. |
| **Table S3** | Primers used for mutation study. |
| **Table S4** | Analysis via Hotspot Wizard 3.0 server. |
| **Table S5** | ACA ELISA assay results. |

**Methods**

**Mutation library Construction of DBAT.**

Using pET-32a-DBAT plasmid as a template, polymerase chain reaction (PCR) amplification was performed using PrimeSTAR^®^ Max DNA polymerase. The PCR amplification protocol consisted of denaturation at 98℃ for 3 min followed by 30 cycles of denaturation at 98℃ for 10 s, annealing at 65℃ for 15 s, extension at 72℃ for 40 s, and a final hold for an additional 10 min at 72℃. The PCR product was purified and was digested at 37℃ for 1 h with *Dpn*Ⅰ to remove the template plasmid and then was transformed into *E. coli* DH5α. Plasmid extracted from positive transformants were sequenced and transformed into *E. coli* BL21 (DE3). Positive recombinant mutants were cultured overnight in 5 mL of Luria–Bertani (LB) medium [1% (w/v) tryptone, 0.5% (w/v) yeast extract, 1% (w/v) NaCl, pH 7.4] containing ampicillin (100 μg/ml) and then were inoculated with 1% seed solution into 50 mL of TB medium containing ampicillin (100 μg/mL) for induction expression. Other procedures were consistent with the whole-cell catalysis as described above.The bioconversion screening reaction occurred at 25℃. The optimal mutant was selected based on the conversion rate of baccatin Ⅲ and the expression level of the mutants.

**Detection of Intracellular Accumulation of Acetyl-CoA on the *In Vivo* Synthesis of Baccatin Ⅲ**

BL21 (DE3)/pET-32a-DBAT^best^ was used as the starting strainto perform *in vivo* fermentation. The fermentation medium used six kinds of medium supplemented with different carbon sources, which were starch, sucrose, glucose, glycerol, lactose, and fructose (Table S2). The bacteria were grown to OD_600_ = 0.6 at 37ºC with 100 μg/mL ampicillin selection, and then expression was induced by adding 0.02 mM IPTG, and then all experimental groups with different carbon sources were incubated at 25ºC for 48 h. At 4, 8, 12, 24, 32, and 48 h, there was a transfer of 1 ml of each expression culture into a 1.5 ml microcentrifuge tube, respectively. The culture supernatants of these samples were used to measure the cell density by spectrophotometer and the yield of baccatin Ⅲ by HPLC. After this, the best carbon source was chosen to further investigate the effect of different levels added (5, 10, 15, 20, and 30 g/L).

**Determination of Acetyl-CoA Accumulation in Different Strains**

*E. coli* strain N05 was reported as an engineered strainwith high acetyl-CoA production. To investigate the acetyl-CoA yield of *E. coli* N05 and *E. coli* BL21 (DE3), ACA ELISA kit was used to detect the acetyl-CoA content in different stages during fermentation. All operations performed according to the manufacturer’s manual. Three experimental groups were set up: BL21 (DE3) with 20 g/L glycerol addition, N05 with 20 g/L glycerol addition and N05 with 5 g/L glycerol addition. Two periods were selected for bacterial cell concentration OD_600_ to reach 0.6 and 1.6.

**Construction and Fermentation of Mutant expressed in N05**

The P*_tac_* promoter was integrated into plasmid pET-32a-DBAT*^mut^* to construct plasmid pET-32a-P*_tac_*-DBAT*^mut^*. The linear backbone of plasmid pET-32a-P*_tac_*-DBAT*^mut^* was amplified by a pair of 5’-phosphorylated primers tac_F/tac_R (see Table S2) containing half of the promoter P*_tac_* separately. The PCR product was purified by the HiPure Gel Pure DNA Micro Kit. After digestion with *Dpn*Ⅰ at 37℃ for 1 h to remove the parent plasmid, the phosphorylated PCR recovery product was ligated for 1 h at 22℃ by T4 DNA ligase and then transformed into *E. coli* strain N05 and BL21 (DE3). TB-10-gly medium (see Table S3), with a final concentration of 10 g/L of glycerol, was utilized for fermentation of the strains. 10-DAB (80 μM) was fed into a 50 ml TB-10-gly medium incubating BL21 (DE3)/pET-32a-P*_tac_*-DBAT*^mut^* and N05/pET-32a-P*_tac_*-DBAT*^mut^*, respectively. All fermentation operations and baccatin Ⅲ detection methods were the same as described.

**BaccatinⅢ Production by Engineered Strain N05S01**

Plasmid pHKTT5b-*s189v*^o^ was mutated from plasmid pHKTT5b-*dbat*^o^ by site-directed mutagenesis as described. The engineered strains BWS01 and N05S01 were constructed as the same as described. BWD01, BWS01 and N05S01 were fermented in 50 mL fresh TB medium, respectively. After adding 80 μM 10-DAB, all cultures were incubated at 25℃ and 150 rpm for 48 h. After fermentation, baccatin Ⅲ yields in culture supernatant were detected by HPLC as described. All assays were carried out in triplicate.


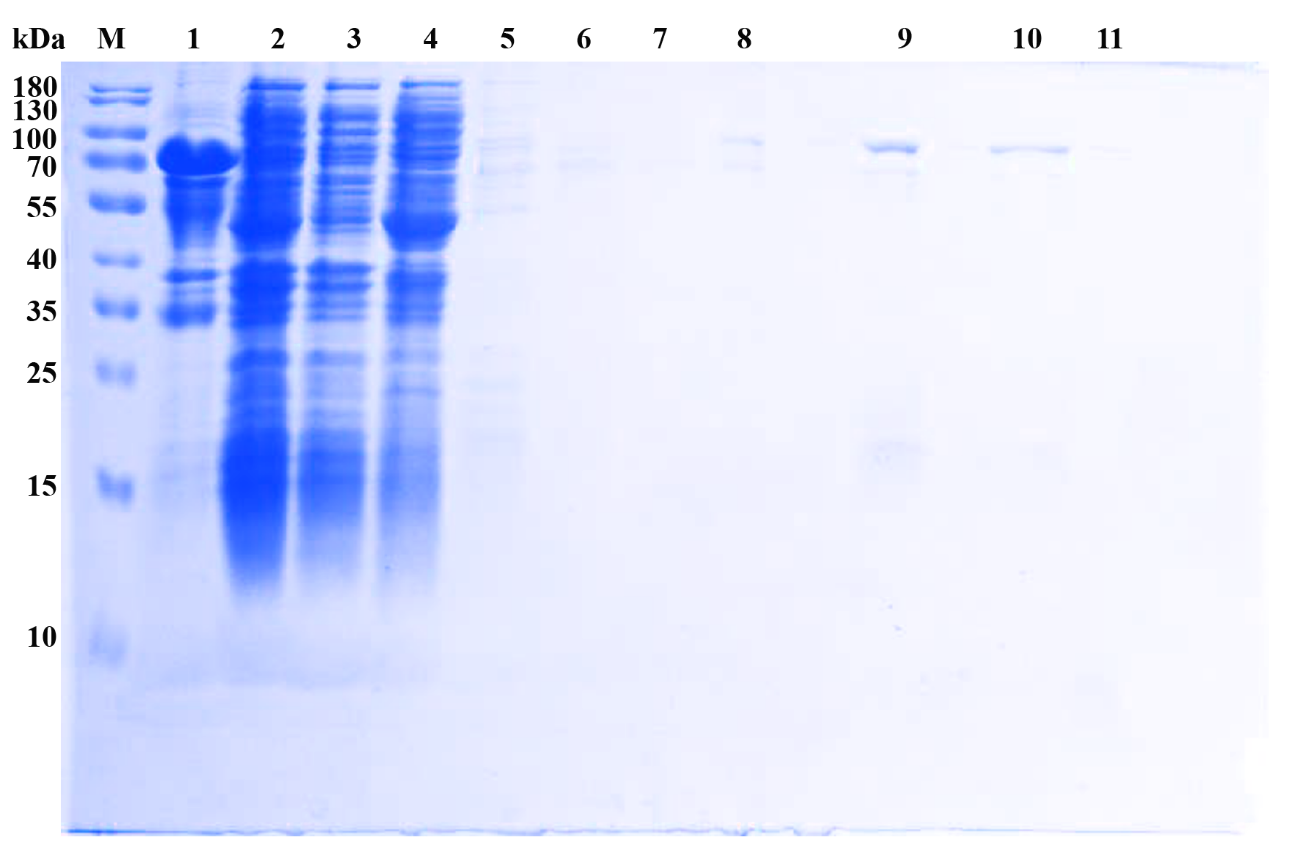


**Fig. S1.**SDS-PAGE analysis of DBAT expression and purification. The target protein DBAT is about 67 kDa.**M** molecular weight markers, **1** precipitation after cell disruption, **2** crude extract,**3-9** filtrate of the purification process, **10-11** purified protein.

**
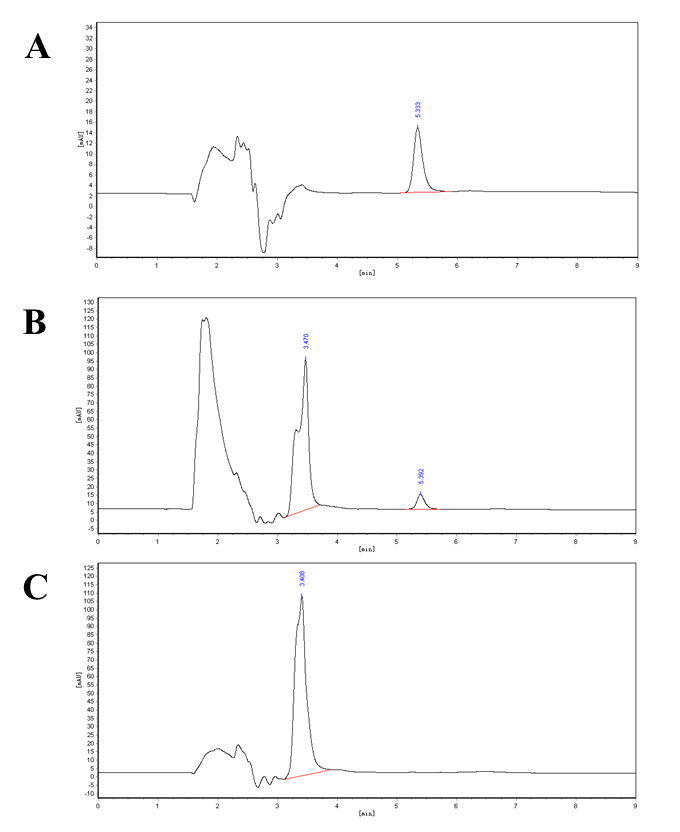
**

**Fig. S2.**The HPLC results of whole-cell bioconversion. **(A**) HPLC result for standard baccatin Ⅲ. (**B**) The results of whole-cell bioconversion to baccatin Ⅲ by adding glucose as an activator. (**C**) The results of whole-cell bioconversion to baccatin Ⅲ without adding glucose.


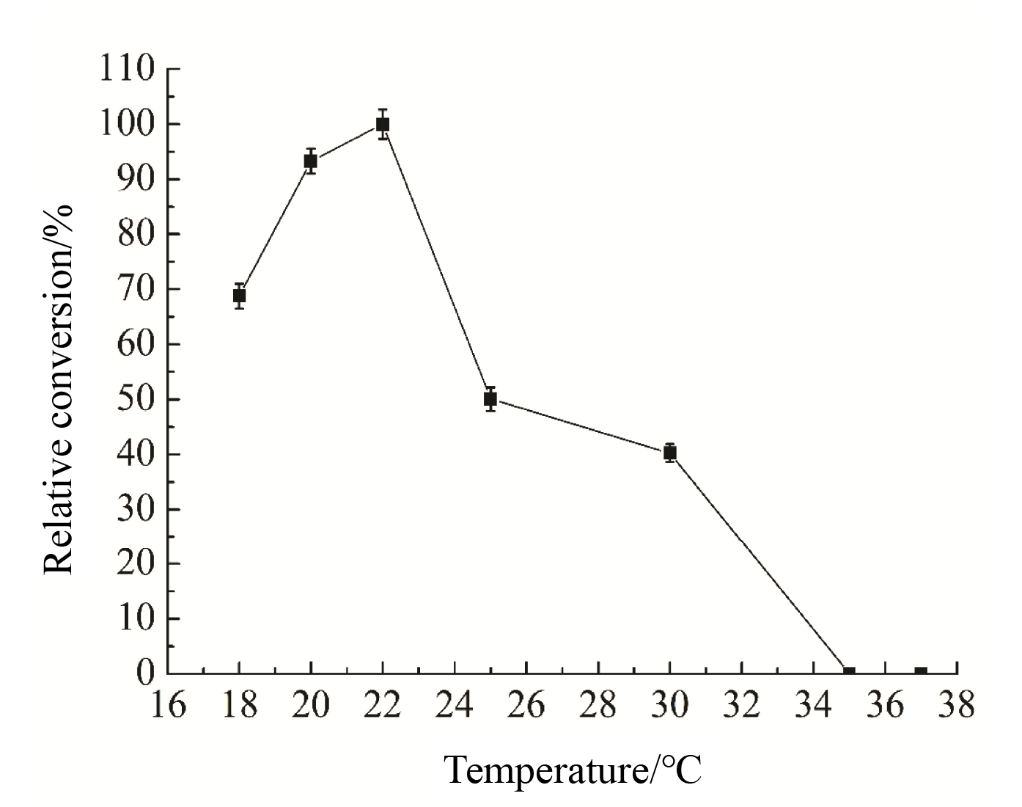


**Fig. S3.**Theeffect of temperature on the production of baccatin Ⅲ by whole-cell bioconversion.


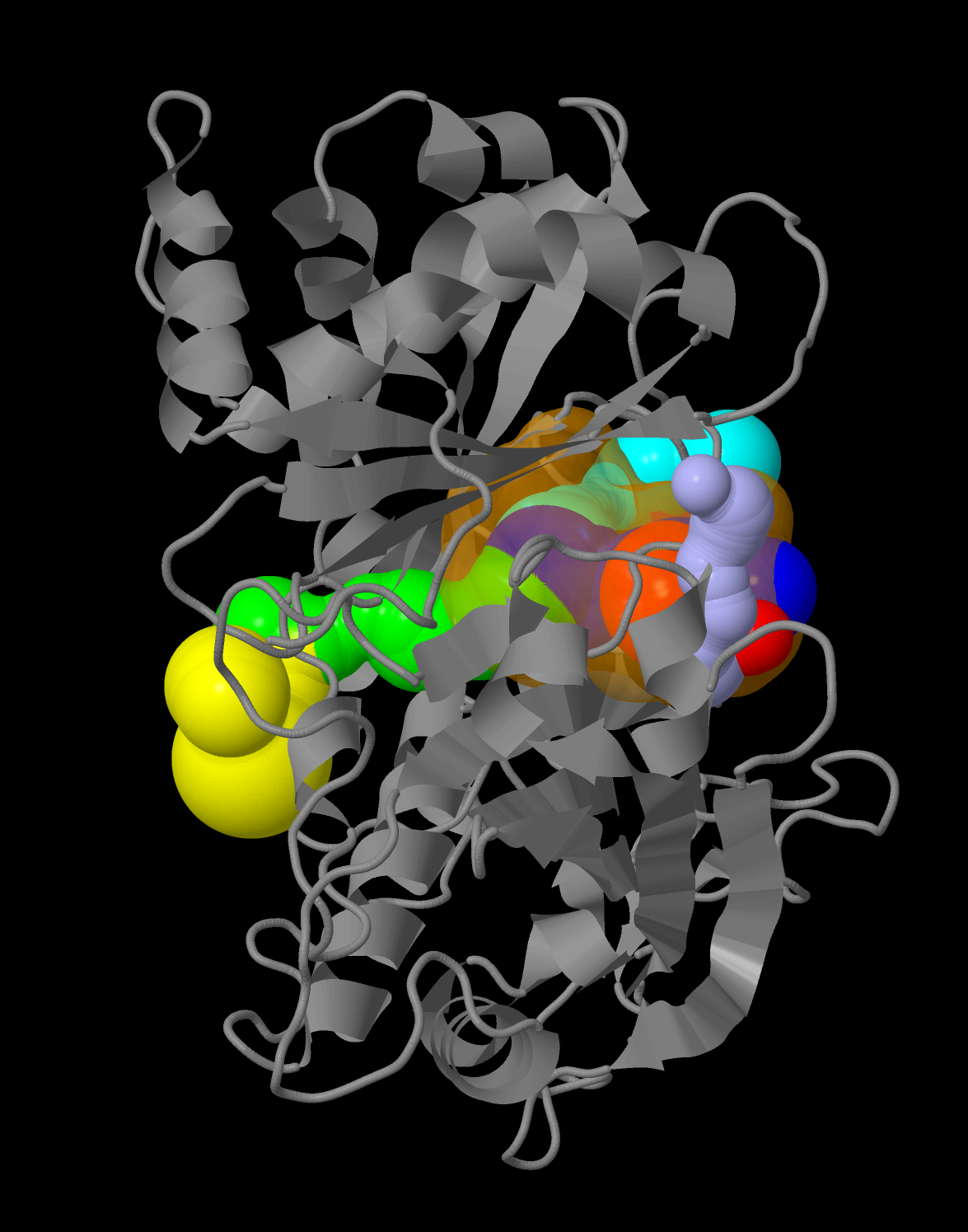


**Fig. S4.** The solvent channel and binding pocket of DBAT.


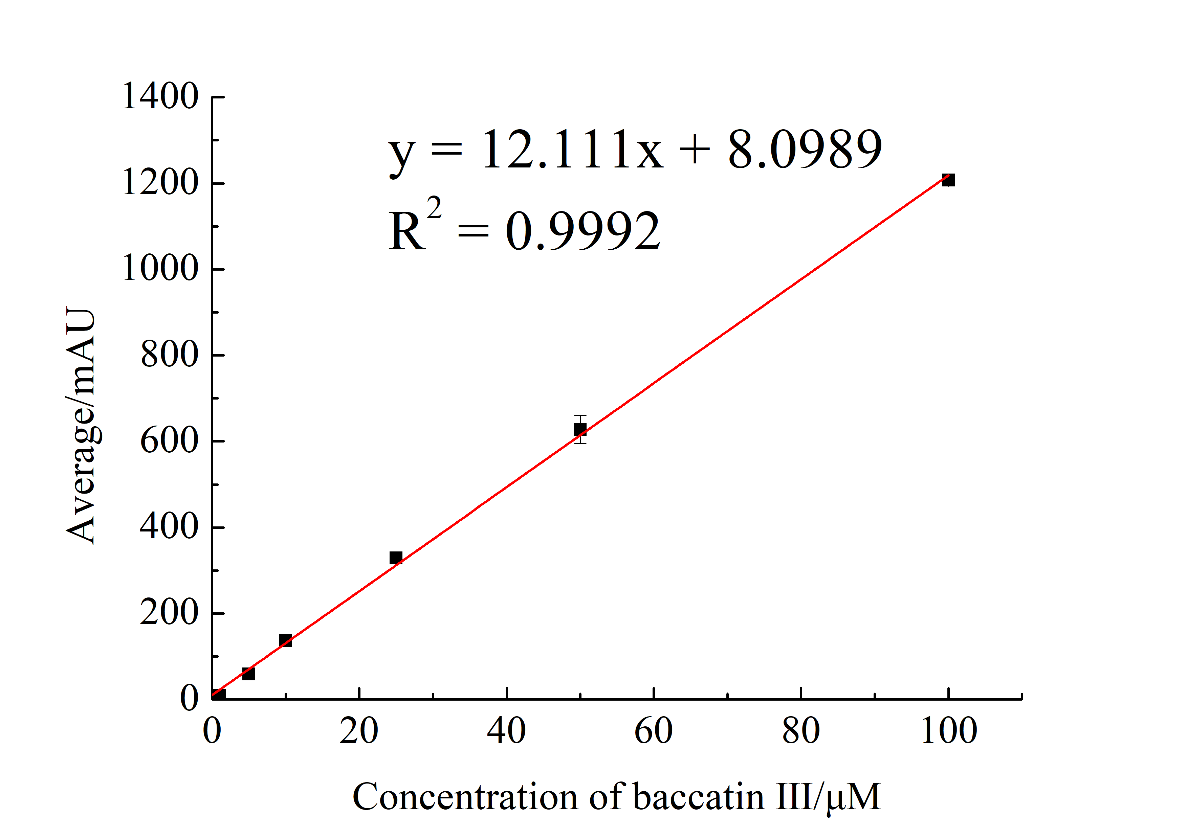


**Fig. S5.**The baccatin Ⅲ standard curve.


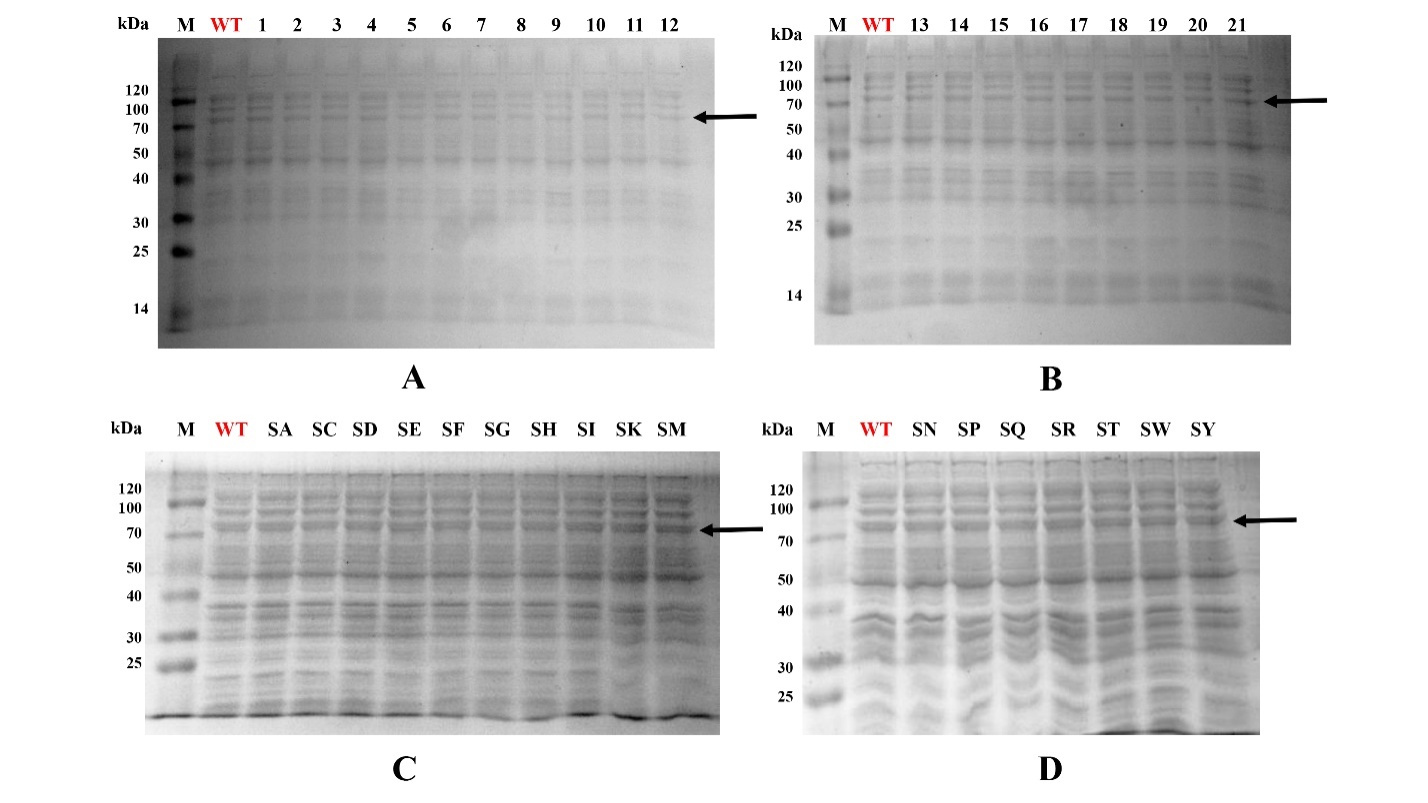


**Fig. S6** The SDS-PAGE results of mutants by staining with Coomassie blue.(**A, B)**SDS-PAGE analysis of “hotspot” mutants, 1-21: P37T,V39A, N42H,I43N,S122D,H123S,E124A,S159F,L168Q,G171C,I175Q,S189L,P37Q,V39L,N42F,I43H,S189V,H123L,S159R,I175N, and S122C. (**C, D**) SDS-PAGE analysis of site-saturated mutants. The band with the black arrow represents the target protein.


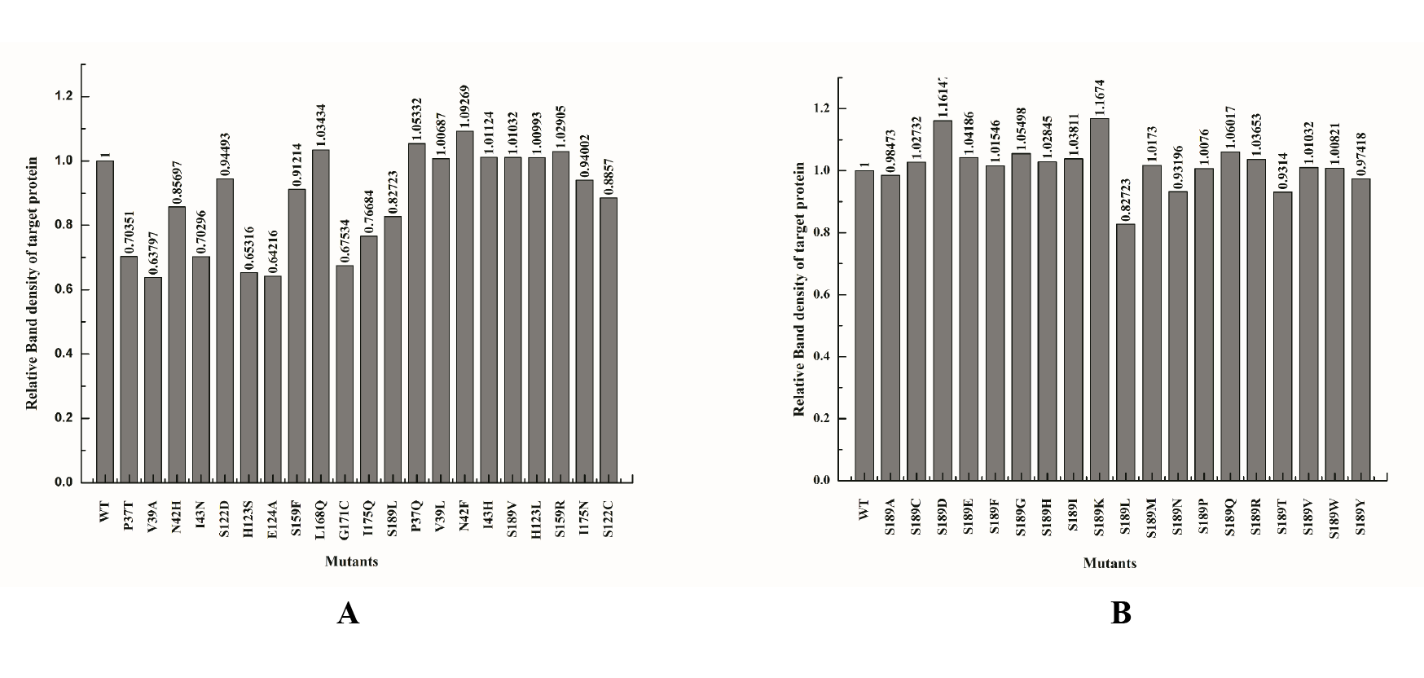


**Fig. S7.**Relative supernatant expression of mutants. (**A**) The expression level results for“hotspot” mutants. (**B**) The expression level results for site-saturated mutants.


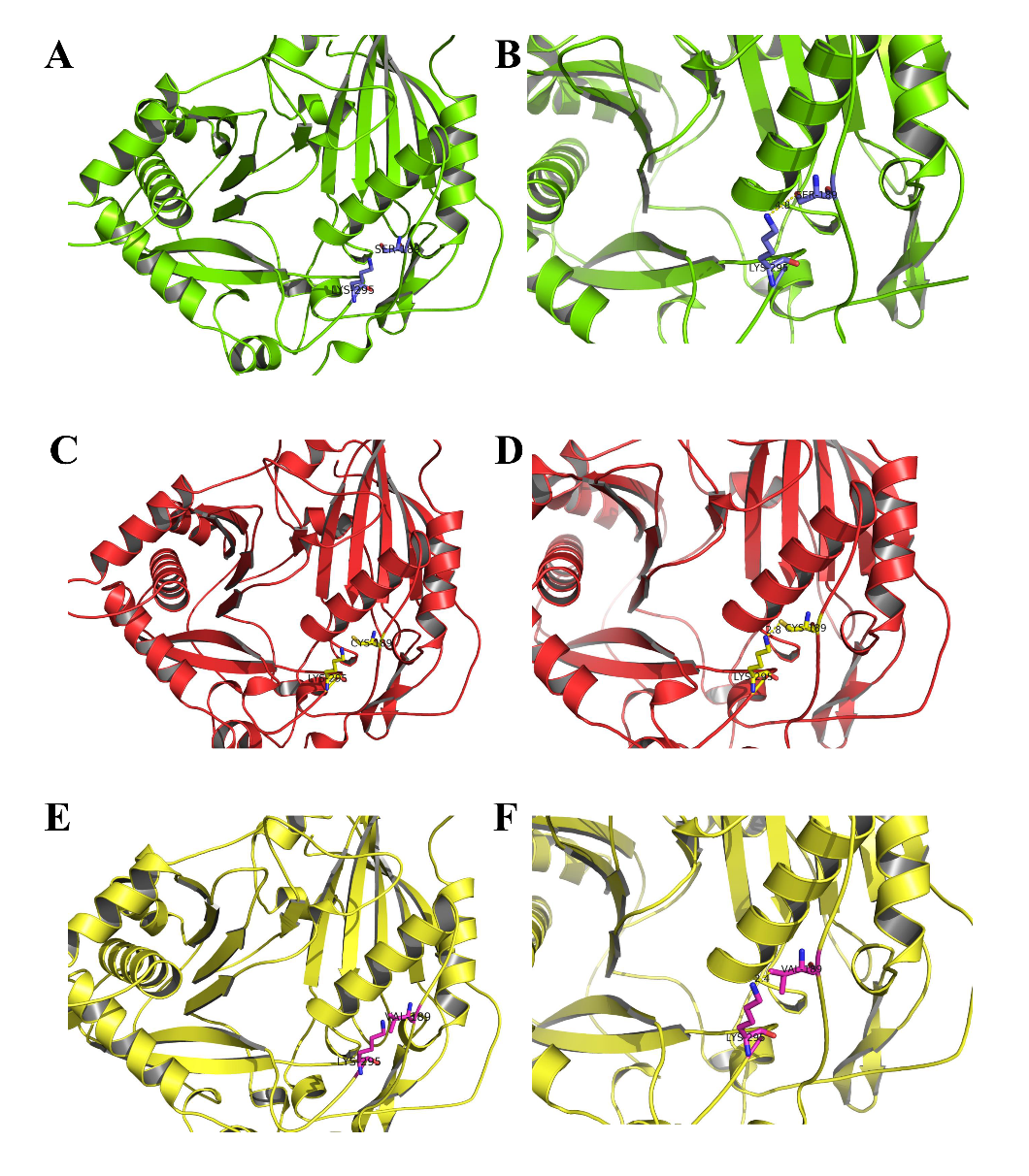


**Fig. S8** Skeleton structure analysis of the S189 mutants.**(A, B)**The distance between Ser189 and the adjacent residue Lys295 in WT is 4.8 Å. The DBAT model was displayed as cartoon style in green, the residue Lys295 and Ser189 were displayed as sticks style in blue. **(C, D)**The hydrogen bond between Cys189 and the adjacent residue Lys295 in DBAT^S189C^, the distance between them is 2.8 Å. The DBAT^S189C^ model was displayed as cartoon style in red, the residue Lys295 and Cys189 were displayed as sticks style in yellow.**(E, F)** The hydrogen bond between Val189 and the adjacent residue Lys295 in DBAT^S189V^, the distance between them is 2.4 Å. The DBAT^S189V^ model was displayed as cartoon style in yellow, the residue Lys295 and Val189 were displayed as sticks style in purple.

**Table S1 Primers used for vectors construction.**

| **Primers** | **Content** | **Reference/sources** |
| --- | --- | --- |
| dbat_optF | ATGGCGGGTTCTACCGAATTCGTTGTTCGTTCTCTGGAACGTGTTATGGT | Optimized *dbat*gene |
| dbat_R | ACGCGTCGACTCAAGGTTTAGTTACATATTTGTTTGTCATGGTTT | Optimized *dbat*gene |
| rbs_dbat_optF | CGGGATCCTTTCGGAATTAAGGAGGTAATAAATATGGCGGGTTCTACC | Optimized *dbat*gene with RBS sequence |
| AHP3 | AACGAGTATCGAGATGGCAC | Diagnostic PCR in *attP*_HK_ integration |
| HKP4 | CATCAACAGCACATTCAGTGG | Diagnostic PCR in *attP*_HK_ integration |
| HKP1 | CATCAACAGCACATTCAGTGG | Diagnostic PCR in *attP*_HK_ integration |
| A67R | GGAGAGGACCTGCCGAATTACTTTTGCAGGATC | Diagnostic PCR in *attP*_HK_ integration and identification of vector construction |
| HK21F | CGTCTTGAGCGATTGTGTAGG | Identification of vector construction |
| tac_F | 5′ -Phos-GGCTCGTATAATGTGTGGAATTGTGAGCG  GATAACAATTC-3′ | pET-32a-P*_tac_*-DBAT^S189V^ |
| tac_R | 5′ -Phos-GATGATTAATTGTCAACCTATAGTGAGTCG  TATTAATTTCG-3′ | pET-32a-P*_tac_*-DBAT^S189V^ |

**Table S2 Formulation of medium.**

| **Medium** | **Formulation** |
| --- | --- |
| TB | 1.2% (w/v) tryptone, 2.4% (w/v) yeast extract, 0.5% (w/v) glycerol, 17 mM KH_2_PO_4_, 72 mM K_2_HPO_4_ |
| TB-glc | 1.2% (w/v) tryptone, 2.4% (w/v) yeast extract, 0.5% (w/v) glucose, 17 mM KH_2_PO_4_, 72 mM K_2_HPO_4_ |
| TB-sta | 1.2% (w/v) tryptone, 2.4% (w/v) yeast extract, 0.5% (w/v) starch, 17 mM KH_2_PO_4_, 72 mM K_2_HPO_4_ |
| TB-suc | 1.2% (w/v) tryptone, 2.4% (w/v) yeast extract, 0.5% (w/v) sucrose, 17 mM KH_2_PO_4_, 72 mM K_2_HPO_4_ |
| TB-fru | 1.2% (w/v) tryptone, 2.4% (w/v) yeast extract, 0.5% (w/v) fructose, 17 mM KH_2_PO_4_, 72 mM K_2_HPO_4_ |
| TB-lac | 1.2% (w/v) tryptone, 2.4% (w/v) yeast extract, 0.5% (w/v) lactose, 17 mM KH_2_PO_4_, 72 mM K_2_HPO_4_ |

**Table S3Primers used for mutation study.**

| **Primer name** | **Sequence (5’→3’,the corresponding mutant amino acid were labeled italic)** |
| --- | --- |
| L168QF | ATATGTGATGGA*CAA*GGAGCAGGCCAGTTTCTT |
| L168QR | TGCTCC*TTG*TCCATCACATATACCATGGCAGAA |
| G171CF | GGACTAGGAGCA*TGC*CAGTTTCTTATAGCCATG |
| G171CR | AAACTG*GCA*TGCTCCTAGTCCATCACATATACC |
| S122DF | GACTACAGTCCT*GAT*CATGAGCAACTACTTTTT |
| S122DR | CTCATG*ATC*AGGACTGTAGTCATCCAAATCTCC |
| H123SF | TACAGTCCTTCA*TCT*GAGCAACTACTTTTTTGT |
| H123SR | TTGCTC*AGA*TGAAGGACTGTAGTCATCCAAATC |
| I175QF | GGCCAGTTTCTT*CAA*GCCATGGGAGAGATGGCA |
| I175QR | CATGGC*TTG*AAGAAACTGGCCTGCTCCTAGTCC |
| E124AF | AGTCCTTCACAT*GCG*CAACTACTTTTTTGTCTT |
| E124AR | TAGTTG*CGC*ATGTGAAGGACTGTAGTCATCCAA |
| S159FF | GTTGTGGGGATG*TTC*TTCTGCCATGGTATATGT |
| S159FR | GCAGAA*GAA*CATCCCCACAACAAAACCTCCACA |
| P37TF | CTTGACAATCTA*ACA*GGGGTGAGAGAAAACATT |
| P37TR | CACCCC*TGT*TAGATTGTCAAGGGTGGAGAGCTG |
| V39AF | AATCTACCAGGG*GCG*AGAGAAAACATTTTTAAC |
| V39AR | TTCTCT*CGC*CCCTGGTAGATTGTCAAGGGTGGA |
| I43NF | GTGAGAGAAAAC*AAT*TTTAACACCTTGTTAGTC |
| I43NR | GTTAAA*ATT*GTTTTCTCTCACCCCTGGTAGATT |
| P37QF | CTTGACAATCTA*CAA*GGGGTGAGAGAAAACATT |
| P37QR | CACCCC*TTG*TAGATTGTCAAGGGTGGAGAGCTG |
| V39LF | AATCTACCAGGG*CTT*AGAGAAAACATTTTTAAC |
| V39LR | TTCTCT*AAG*CCCTGGTAGATTGTCAAGGGTGGA |
| N42FF | GGGGTGAGAGAA*TTC*ATTTTTAACACCTTGTTA |
| N42FR | AAT*GAA*TTCTCTCACCCCTGGTAGATTGTCAAG |
| I43HF | GTGAGAGAAAAC*CAC*TTTAACACCTTGTTAGTC |
| I43HR | GTTAAA*GTG*GTTTTCTCTCACCCCTGGTAGATT |
| S122CF | GACTACAGTCCT*TGT*CATGAGCAACTACTTTTT |
| S122CR | CTCATG*ACA*AGGACTGTAGTCATCCAAATCTCC |
| H123LF | TACAGTCCTTCA*CTC*GAGCAACTACTTTTTTGT |
| H123LR | TTGCTC*GAG*TGAAGGACTGTAGTCATCCAAATC |
| S159RF | GTTGTGGGGATG*AGA*TTCTGCCATGGTATATGT |
| S159RR | GCAGAA*TCT*CATCCCCACAACAAAACCTCCACA |
| G171AF | GGACTAGGAGCA*GCC*CAGTTTCTTATAGCCATG |
| G171AR | AAACTG*GGC*TGCTCCTAGTCCATCACATATACC |
| I175NF | GGCCAGTTTCTT*AAC*GCCATGGGAGAGATGGCA |
| I175NR | CATGGC*GTT*AAGAAACTGGCCTGCTCCTAGTCC |
| S189LF | ATTAAGCCCTCC*TTG*GAGCCAATATGGAAGAGA |
| S189LR | TGGCTC*CAA*GGAGGGCTTAATCTCTCCCCTTGC |
| S189VF | ATTAAGCCCTCC*GTG*GAGCCAATATGGAAGAGA |
| S189VR | TGGCTC*CAC*GGAGGGCTTAATCTCTCCCCTTGC |
| S189FF | ATTAAGCCCTCC*TTC*GAGCCAATATGGAAGAGA |
| S189FR | TGGCTC*GAA*GGAGGGCTTAATCTCTCCCCTTGC |
| S189YF | ATTAAGCCCTCC*TAC*GAGCCAATATGGAAGAGA |
| S189YR | TGGCTC*GTA*GGAGGGCTTAATCTCTCCCCTTGC |
| S189CF | ATTAAGCCCTCC*TGC*GAGCCAATATGGAAGAGA |
| S189CR | TGGCTC*GCA*GGAGGGCTTAATCTCTCCCCTTGC |
| S189WF | ATTAAGCCCTCC*TGG*GAGCCAATATGGAAGAGA |
| S189WR | TGGCTC*CCA*GGAGGGCTTAATCTCTCCCCTTGC |
| S189PF | ATTAAGCCCTCC*CCA*GAGCCAATATGGAAGAGA |
| S189PR | TGGCTC*TGG*GGAGGGCTTAATCTCTCCCCTTGC |
| S189HF | ATTAAGCCCTCC*CAT*GAGCCAATATGGAAGAGA |
| S189HR | TGGCTC*ATG*GGAGGGCTTAATCTCTCCCCTTGC |
| S189QF | ATTAAGCCCTCC*CAA*GAGCCAATATGGAAGAGA |
| S189QR | TGGCTC*TTG*GGAGGGCTTAATCTCTCCCCTTGC |
| S189RF | ATTAAGCCCTCC*CGA*GAGCCAATATGGAAGAGA |
| S189RR | TGGCTC*TCG*GGAGGGCTTAATCTCTCCCCTTGC |
| S189IF | ATTAAGCCCTCC*ATC*GAGCCAATATGGAAGAGA |
| S189IR | TGGCTC*GAT*GGAGGGCTTAATCTCTCCCCTTGC |
| S189MF | ATTAAGCCCTCC*ATG*GAGCCAATATGGAAGAGA |
| S189MR | TGGCTC*CAT*GGAGGGCTTAATCTCTCCCCTTGC |
| S189TF | ATTAAGCCCTCC*ACA*GAGCCAATATGGAAGAGA |
| S189TR | TGGCTC*TGT*GGAGGGCTTAATCTCTCCCCTTGC |
| S189NF | ATTAAGCCCTCC*AAC*GAGCCAATATGGAAGAGA |
| S189NR | TGGCTC*GTT*GGAGGGCTTAATCTCTCCCCTTGC |
| S189KF | ATTAAGCCCTCC*AAG*GAGCCAATATGGAAGAGA |
| S189KR | TGGCTC*CTT*GGAGGGCTTAATCTCTCCCCTTGC |
| S189AF | ATTAAGCCCTCC*GCA*GAGCCAATATGGAAGAGA |
| S189AR | TGGCTC*TGC*GGAGGGCTTAATCTCTCCCCTTGC |
| S189DF | ATTAAGCCCTCC*GAC*GAGCCAATATGGAAGAGA |
| S189DR | TGGCTC*GTC*GGAGGGCTTAATCTCTCCCCTTGC |
| S189EF | ATTAAGCCCTCC*GAA*GAGCCAATATGGAAGAGA |
| S189ER | TGGCTC*TTC*GGAGGGCTTAATCTCTCCCCTTGC |
| S189GF | ATTAAGCCCTCC*GGA*GAGCCAATATGGAAGAGA |
| S189GR | TGGCTC*TCC*GGAGGGCTTAATCTCTCCCCTTGC |

**Table S4 Analysis via Hotspot Wizard 3.0 server.**

| **Amino acid** | **Mutational landscape (100)** | **Amino acid frequency** |
| --- | --- | --- |
| Pro37 | Thr | Gln (18%) |
| Val39 | Ala | Leu (43%) |
| Asn42 | His | Phe (29%) |
| Ile43 | Asn | His (17%) |
| Ser122 | Asp | Cys (25%) |
| His123 | Ser | Leu (33%) |
| Glu124 | Ala | -- |
| Ser159 | Phe | Arg (22%) |
| Leu168 | Gln | -- |
| Gly171 | Cys | -- |
| Ile175 | Gln | Asn (29%) |
| Ser189 | Leu | Val (28%) |

--: Non-mutation was the best choice of the amino acid frequency of this residue

**Table S5 ACA ELISA assay results.**

|  | **OD_600_=0.6**  **(ng/mL)** | **OD_600_=1.6**  **(ng/mL)** | **Change in content during logarithmic phase（±）** |
| --- | --- | --- | --- |
| BL21 (DE3)-20 g/L | 49.09 ± 0.25 | 47.26 ± 0.86 | -1.83 |
| N05-20 g/L | 40.56 ± 0.74 | 47.82 ± 1.64 | +7.26 |
| N05-5 g/L | 41.95 ± 1.72 | 46.48 ± 0.24 | +4.53 |
